# Product ion distributions for the reactions of NO^+^ with some physiologically significant volatile organosulfur and organoselenium compounds obtained using a selective reagent ionization time-of-flight mass spectrometer

**DOI:** 10.1002/rcm.6947

**Published:** 2014-06-18

**Authors:** Paweł Mochalski, Karl Unterkofler, Patrik Španěl, David Smith, Anton Amann

**Affiliations:** 1 Breath Research Institute of the University of InnsbruckRathausplatz 4, A-6850, Dornbirn, Austria; 2 Vorarlberg University of Applied SciencesHochschulstr. 1, A-6850, Dornbirn, Austria; 3 Univ.-Clinic for Anesthesia and Intensive Care, Innsbruck Medical UniversityAnichstr, 35, A-6020, Innsbruck, Austria; 4 J. Heyrovský Institute of Physical Chemistry, Academy of Sciences of the Czech RepublicDolejškova 3, 18223, Prague 8, Czech Republic; 5 Institute for Science and Technology in Medicine, Medical School, Keele UniversityThornburrow Drive, Hartshill, Stoke-on-Trent, ST4 7QB, UK

## Abstract

**RATIONALE:**

The reactions of NO^+^ with volatile organic compounds (VOCs) in Selective Reagent Ionization Time-of-Flight Mass Spectrometry (SRI-TOF-MS) reactors are relatively poorly known, inhibiting their use for trace gas analysis. The rationale for this product ion distribution study was to identify the major product ions of the reactions of NO^+^ ions with 13 organosulfur compounds and 2 organoselenium compounds in an SRI-TOF-MS instrument and thus to prepare the way for their analysis in exhaled breath, in skin emanations and in the headspace of urine, blood and cell and bacterial cultures.

**METHODS:**

Product ion distributions have been investigated by a SRI-TOF-MS instrument at an E/N in the drift tube reactor of 130 Td for both dry air and humid air (4.9% absolute humidity) used as the matrix gas. The investigated species were five monosulfides (dimethyl sulfide, ethyl methyl sulfide, methyl propyl sulfide, allyl methyl sulfide and methyl 5-methyl-2-furyl sulfide), dimethyl disulfide, dimethyl trisulfide, thiophene, 2-methylthiophene, 3-methylthiophene, methanethiol, allyl isothiocyanate, dimethyl sulfoxide, and two selenium compounds – dimethyl selenide and dimethyl diselenide.

**RESULTS:**

Charge transfer was seen to be the dominant reaction mechanism in all reactions under study forming the M^+^ cations. For methanethiol and allyl isothiocyanate significant fractions were also observed of the stable adduct ions NO^+^M, formed by ion-molecule association, and [M–H]^+^ ions, formed by hydride ion transfer. Several other minor product channels are seen for most reactions indicating that the nascent excited intermediate (NOM)^+^* adduct ions partially fragment along other channels, most commonly by the elimination of neutral CH_3_, CH_4_ and/or C_2_H_4_ species that are probably bound to an NO molecule. Humidity had little effect on the product ion distributions.

**CONCLUSIONS:**

The findings of this study are of particular importance for data interpretation in studies of volatile organosulfur and volatile organoselenium compounds employing SRI-TOF-MS in the NO^+^ mode. © 2014 The Authors. *Rapid Communications in Mass Spectrometry* published by John Wiley & Sons Ltd.

There is considerable evidence that volatile organic compounds (VOCs) produced and then partially released by the human body have great potential for diagnosis in physiology and medicine.[1–6] The emission of such compounds may result from normal human metabolism as well as from pathophysiological disorders, bacterial or mycotic processes[7–11] or exposure to environmental contaminants.[12] As human-specific chemical fingerprints, VOCs can provide non-invasive and real-time information on infections, metabolic disorders and the progression of therapeutic intervention. Apart from medical applications, VOC emissions by humans and their detection may potentially be used in safety and security applications by improving the early location of entrapped victims and, thereby, the success of search and rescue operations (USaR).[13,14]

Volatile sulfur compounds (VSCs), which are almost invariably organic, are an important class of compounds frequently reported to be present in human tissues and fluids, as shown in Table 1. A number of species from this chemical family have been found in human urine,[13,15,16] blood,[17] and exhaled breath.[15,17–19] Several VSCs have been reported to be released by *in vitro* human cell lines,[20] including *E. coli* that releases methanethiol, dimethyl sulfide and hydrogen sulfide (strictly speaking not a VOC) that have been quantified and their emissions followed in real time.[21,22] As members of fluid/tissue-specific chemical fingerprints, sulfur compounds have been proposed to be markers of several disease conditions. In particular their elevated breath levels have been attributed to halitosis,[23] liver diseases,[24–26] schizophrenia,[27] and lung cancer.[28,29] In therapeutic monitoring, breath OCS has been suggested to be a marker of organ rejection after lung transplantation,[30] whereas breath CS_2_ has been evidenced as an indicator of disulfiram ingestion.[31]

**Table 1:** Volatile organosulfur and organoselenium compounds under study and their occurrence in human fluids and tissues

Compound	CAS	Found in tissue/fluid
Dimethyl sulfide	75-18-3	a) Breath[15,17–19,23,57]
		b) Blood[17,57,58]
		c) Urine[13,16]
		d) Skin emanation[59]
		e) Released by cell cultures[20]
Ethyl methyl sulfide	624-89-5	a) Breath[15,17]
		b) Blood[17]
		c) Urine[15]
		d) Released by cell cultures[20]
Methyl propyl sulfide	3877-15-4	a) Breath[15,17,57]
		b) Blood[17,57]
Allyl methyl sulfide	10152-76-8	a) Breath[15,17,19,57]
		b) Blood[17,57]
		c) Skin emanation[59]
Methyl 5-methyl-2-furyl sulfide	13678-59-6	a) Released by cell cultures[20]
Dimethyl disulfide	624-92-0	a) Breath[18,19]
		b) Urine[16,60,61]
		c) Skin emanation[62]
Dimethyl trisulfide	3658-80-8	a) Urine[13,16,60,61]
Thiophene	110-02-1	a) Blood[17,57]
2-Methylthiophene	554-14-3	a) Breath[15]
		b) Urine[15]
3-Methylthiophene	616-44-4	a) Breath[15,19]
		b) Blood[17]
		c) Released by cell cultures[20]
Methanethiol	74-93-1	a) Breath[15,18,23]
		b) Blood[23]
		c) Urine[13,15,60]
		d) Skin emanation[62]
Allyl isothiocyanate	57-06-7	a) Urine[13,61]
Dimethyl sulfoxide	67-68-5	a) Urine[63]
Dimethyl selenide	593-79-3	a) Breath[15,17,19]
		b) Blood[17]
		c) Urine[15,64]
Dimethyl diselenide	7101-31-7	a) Urine[64]

CAS: Chemical Abstracts Service number.

The main obstacle limiting the broader clinical application of VSCs is insufficient understanding of their origin and metabolic fate. Nevertheless, several sources of their presence in humans can be indicated. These include (i) systemic metabolization of sulfur-containing amino acids (methionine, cysteine);[23], (ii) bacterial activity in the oral cavity and colon;[23,32], and (iii) dietary intake.[33] The analysis of VSCs is challenging and poses several analytical problems[34] largely due to their highly reactive nature manifest in their absorptive, adsorptive and photooxidative properties. Thus, significant losses of VSCs can occur during sample processing and analysis, which distort the original chemical fingerprint. Within this framework, the reliable identification and quantification of VSCs is very important for their potential use in biomedical application.

Selenium is a vital trace element in the human organism that can readily substitute the sulfur in proteins. In particular, it forms the two special amino acids selenomethionine and selenocysteine, which are believed to be natural antioxidants capable of neutralizing reactive oxygen species (ROS). Since the metabolism of these amino acids should be analogous to that of their sulfur counterparts, monitoring of volatile selenium compounds present in human tissues/fluids may provide evidence of selenium deficiency or excessive intake. Thus, quantitative data on volatile selenium species would be a valuable addition to information on volatile sulfur species. It is also interesting to note that some organoselenium compounds are released by plants cultivated in a selenium-rich medium.[35]

Real-time analytical techniques such as proton-transfer reaction mass spectrometry (PTR-MS)[36,37] and selected ion flow tube mass spectrometry (SIFT-MS)[38,39] are particularly attractive in the context of the analysis of VSCs since their use effectively eliminate the losses of analytes that can occur by sample collection and storage and sample pre-concentration phases of analysis. PTR-MS is a well-established analytical tool for the detection and quantification of VOCs in biological, medical and environmental studies.[40–43] This stems from its versatility, excellent sensitivity (low pptv concentration levels), and real-time response. Recently, the analytical power of the PTR-MS technique has been notably enhanced by (i) the application of a Time-of-Flight (TOF) mass filter creating PTR-TOF-MS, and (ii) employment of additional precursor ions such as NO^+^, O_2_^+^, and Kr^+^ instead of the usual H_3_O^+^ (creating a Selective Reagent Ionization Time-of-Flight Mass Spectrometry (SRI-TOF-MS) instrument).[44–46] The former improvement notably increased the instrument resolving power (up to 5000 m/Δm) and thus allows the separation of isobaric ions.

The primary goal of the present work was to investigate the product ion distributions for the reactions of NO^+^ ions with 13 volatile sulfur compounds and 2 volatile selenium compounds that are involved in human physiology using a SRI-TOF-MS instrument. The reactions of NO^+^ with VOCs in SRI-TOF-MS reactors are relatively poorly understood, although experiments have now begun with a very recent study of the reactions of NO^+^ with a series of aldehydes of biogenic significance.[47] This undesirable situation is particularly true for volatile sulfur compounds. An interesting aspect of NO^+^ ion chemistry is that its reactions with VOCs are diverse and include charge transfer, hydride ion (H^–^) transfer, hydroxide ion (OH^–^) transfer, alkoxide ion (OR^–^) transfer and NO^+^ ion-molecule association.[38] Moreover, different chemical classes of VOCs undergo characteristic reaction processes with NO^+^ that in some cases help to separate structural isomers. The VSCs chosen for the present study were selected on the basis of their reported presence in human urine, breath, blood and skin emanation.

## EXPERIMENTAL

### Materials and standard mixtures

Single-compound calibration mixtures were prepared from pure liquid substances. The reference substances with purities ranging from 95 to 99.9% were purchased from Sigma-Aldrich (Vienna, Austria), CHEMOS GmbH (Regenstauf, Germany), SAFC (Vienna, Austria) and Fluka (Buchs, Switzerland). The specific compound purities are given in Table 2 to assist interpretation of the observed product ion distributions of the reactions.

**Table 2:** Product ion distributions for the reactions of NO^+^ with volatile sulfur and selenium compounds under dry and humid (AH 4.9%) air carrier gas in the SRI-TOF-MS instrument at a specific E/N of 130 Td. Uncertain neutral products for the reactions are indicated by bracketing. In the case of VSCs the ^34^S isotopologue ions were not taken into consideration in the calculations of percentages

Compound	Formula	Purity [%]	Reaction channel	Dry air [%]	Humid air [%]	Measured m/z [Th]	Expected m/z [Th]	Error [mTh]
Dimethyl sulfide	C_2_H_6_S	99.9	C_2_H_6_S^+^ + NO	91	94	62.0223	62.0185	3.8
			CH_3_S^+^ + (CH_3_NO)	9	6	46.9951	46.9950	0.1
Ethyl methyl sulfide	C_3_H_8_S	96	C_3_H_8_S^+^ + NO	42	44	76.0373	76.0342	3.2
			C_2_H_5_S^+^ + (CH_3_NO)	25	21	61.0129	61.0107	2.3
			C_2_H_4_S^+^ + (CH_4_ + NO)	1.7	1.5	60.0053	60.0028	2.5
			CH_4_S^+^ + (C_2_H_4_ + NO)	31	35	48.0047	48.0034	1.3
Methyl propyl sulfide	C_4_H_10_S	98	C_4_H_10_S^+^ + NO	20	25	90.0534	90.0497	3.6
			C_3_H_7_S^+^ + (CH_3_NO)	10	10	75.0293	75.0263	3.0
			C_2_H_5_S^+^ + (C_2_H_4_ + HNO)	5.4	5.1	61.0128	61.0107	2.2
			CH_5_S^+^ + (C_3_H_5_NO)	39	36	49.012	49.0107	1.4
			CH_4_S^+^ + (C_3_H_6_ + NO)	25	22	48.0047	48.0034	1.3
Allyl methyl sulfide	C_4_H_8_S	98	C_4_H_8_S^+^ + NO	85	87	88.0396	88.0342	5.4
			C_3_H_5_S^+^ + (CH_3_NO)	12	11	73.0151	73.0107	4.4
			C_3_H_4_S^+^ + (CH_4_ + NO)	0.8	0.6	72.0071	72.0028	4.3
			C_2_H_4_S^+^ + (C_2_H_4_ + NO)	1.7	1.6	60.0063	60.0028	3.5
Dimethyl disulfide	C_2_H_6_S_2_	99	C_2_H_6_S_2_^+^ + NO	93	91	93.9974	93.9906	6.8
			CH_3_S_2_^+^ + (CH_3_NO)	0.7	0.5	78.9799	78.9671	12.8
			C_2_H_6_S^+^ + (SNO)	6.5	8.3	62.0263	62.0185	7.8
Dimethyl trisulfide	C_2_H_6_S_3_	98	C_2_H_6_S_3_^+^ + NO	86	87	125.9733	125.9627	10.7
			Unknown	2.1	2.5	94.9696		
			C_2_H_6_S_2_^+^ + (SNO)	0.9	0.9	93.9969	93.9906	6.4
			CH_4_S_2_^+^ + (CH_2_SNO)	2.6	2.0	79.9807	79.9749	5.8
			CH_3_S_2_^+^ + (CH_3_S + NO)	2.2	2.7	78.9737	78.9671	6.6
			CH_2_S_2_^+^ + (CH_3_SH + NO)	1.9	1.5	77.9646	77.9593	5.4
			C_2_H_5_S^+^ + (SS + HNO)	3.6	2.8	61.0141	61.0107	3.5
Thiophene	C_4_H_4_S	99	C_4_H_4_S^+^ + NO	100	100	84.0072	84.0029	4.3
2-Methylthiophene	C_5_H_6_S	98	C_5_H_6_S^+^ + NO	97	98	98.0239	98.0185	5.4
			C_5_H_5_S^+^ + HNO	2.5	2.2	97.0197	97.0107	9.0
3-Methylthiophene	C_5_H_6_S	98	C_5_H_6_S^+^ + NO	98	98	98.0252	98.0185	6.7
			C_5_H_5_S^+^ + HNO	2.5	2.3	97.0192	97.0107	8.5
Methyl 5-methyl-2-furyl sulfide	C_6_H_8_OS	99	C_6_H_8_OS^+^ + NO	92	91	128.0335	128.0291	4.5
			C_5_H_5_OS^+^ + (CH_3_NO)	4.5	5.1	113.0194	113.0056	13.8
			Unknown	3.5	3.9	97.0281		
Methanethiol	CH_3_SH	99	CH_3_SH · NO^+^	6.4	8.1	78.0055	78.0008	4.7
			CH_3_SH^+^ + NO	82	82	48.0048	48.0028	2.0
			CH_3_S^+^ + HNO	7.0	4.7	46.9947	46.9950	0.3
			CHS^+^ + (H_2_ + HNO)	4.1	5.1	44.9807	44.9794	1.4
Allyl isothiocyanate	C_4_H_5_NS	95	C_4_H_5_NS · NO^+^	9.2	10.0	129.0224	129.0117	10.7
			C_4_H_5_NS^+^ + NO	71	69	99.0204	99.0138	6.7
			C_4_H_4_NS^+^ + HNO	12	12	98.0131	98.0059	7.2
			C_2_H_2_NS^+^ + (C_2_H_3_ + NO)	1.8	1.6	72.0040	71.9903	13.7
			C_3_H_5_^+^ + (CNS + NO)	6.1	7.8	41.0398	41.0386	1.3
Dimethyl sulfoxide	C_2_H_6_OS	99.9	C_2_H_6_OS^+^ + NO	81	81	78.01846	78.0134	5.0
			C_1_H_3_OS^+^ + (CH_3_NO)	13	13	62.9938	62.9899	3.9
			C_2_H_5_S^+^ + HNO_2_	5.9	6.2	61.0149	61.0107	4.3
Dimethyl selenide	C_2_H_6_Se	99	C_2_H_6_Se^+^ + NO	99	99	-	-	-
			CH_3_Se^+^ + (CH_3_NO)	1.2	1.0	-	-	-
			C_2_H_6_^82^Se^+^ + NO	8.5	8.6	111.9727	111.9637	9.0
			C_2_H_6_^80^Se^+^ + NO	49	49	109.9723	109.9635	8.8
			C_2_H_6_^78^Se^+^ + NO	24	24	107.9728	107.9643	8.5
			C_2_H_6_^77^Se^+^ + NO	7.8	7.8	106.9756	106.9669	8.7
			C_2_H_6_^76^Se^+^ + NO	9.3	9.3	105.9745	105.9662	8.3
			C_2_H_6_^74^Se^+^ + NO	0.9	0.9	103.9779	103.9695	8.4
			CH_3_^80^Se^+^ + (CH_3_NO)	0.3	0.3	94.9505	94.9400	10.0
			CH_3_^78^Se^+^ + (CH_3_NO)	0.6	0.5	92.9508	92.9408	10.0
Dimethyl diselenide	C_2_H_6_Se_2_	96	C_2_H_6_Se_2_^+^ + NO	99	99	-	-	-
			CH_3_Se_2_^+^ + (CH_3_NO)	0.8	0.7	-	-	-
			C_2_H_6_^80^Se^82^Se^+^ + NO	8.6	8.6	191.8952	191.8802	15.0
			C_2_H_6_^80^Se_2_^+^/C_2_H_6_^78^Se^82^Se^+^ + NO	29	30	189.8944	189.8800	14.4
			C_2_H_6_^78^Se^80^Se^+^/C_2_H_6_^76^Se^82^Se^+^ + NO	26	26	187.8956	187.8808	14.8
			C_2_H_6_^77^Se^80^Se^+^ + NO	7.9	8.0	186.8979	186.8834	14.5
			C_2_H_6_^78^Se_2_^+^/C_2_H_6_^76^Se^80^Se^+^ + NO	16	16	185.8972	185.8821	15.1
			C_2_H_6_^77^Se^78^Se^+^ + NO	3.8	3.7	184.8986	184.8842	14.4
			C_2_H_6_^76^Se^78^Se^+^/C_2_H_6_^74^Se^80^Se^+^ + NO	6.0	6.0	183.8993	183.8835	15.8
			C_2_H_6_^76^Se^77^Se^+^ + NO	1.5	1.5	182.9012	182.8861	15.1
			C_2_H_6_^76^Se_2_^+^/C_2_H_6_^74^Se^78^Se^+^ + NO	1.3	1.3	181.9003	181.8854	14.9
			CH_3_^80^Se_2_^+^/CH_3_^78^Se^82^Se^+^ + (CH_3_NO)	0.2	0.2	174.8724	174.8565	15.9
			CH_3_^78^Se^80^Se^+^/CH_3_^76^Se^82^Se^+^ + (CH_3_NO)	0.2	0.2	172.8700	172.8594	10.6

The preparation of gaseous standard mixtures was performed in a similar way to the procedures outlined in our recent article.[47] For each compound the product ion distribution was investigated using three distinct concentrations of each analyte in high-purity air at a partial pressure variously ranging approximately from 20 to 180 ppbv and at two different absolute humidity levels of essentially 0 and 4.9%.

### SRI-TOF-MS analysis

The NO^+^ reactions were studied using a model 8000 SRI-TOF-MS instrument (Ionicon Analytik, Innsbruck, Austria), a variant of the familiar PTR-MS instruments.[45,48] The NO^+^ reagent ions were produced by charging the hollow cathode discharge ion source with high-purity dry air.[38,49] The settings of the ion source were chosen as follows: ion source current 5 mA, source voltage (U_s_) 20 V, source-out voltage (U_so_) 70 V, and source valve opening 40%. With these settings the major impurity ions, as detected downstream by the SRI-TOF-MS instrument, were H_3_O^+^, O_2_^+^, and NO_2_^+^ at relative levels (parasitic ion/NO^+^) of 0.3–0.6%, 1–1.5%, and 1–2%, respectively, in the air carrier/buffer gas. The NO^+^/volatile reactions occurred in the sample gases in the drift tube at a total pressure of 2.3 mbar and a gas temperature of 60 °C. The voltage along the drift section was set to 600 V leading to an E/N ratio of approximately 130 Td.[50] The E/N value at which the experiments/analyses were carried out may have an important influence on the rate constants and the product ions distributions for these NO^+^ reactions, a point referred to later in the Results section.

The mass spectra over the range from approximately *m/z* 2.7 to 500 were acquired for a period of 30 s by co-adding 750 000 single 40-µs long TOF-MS extractions recorded at a sampling frequency of 1/Δt = 10 GHz. The actual mass resolution obtained from the detected peaks was ≈ 4000 at *m/z* 100. The mass calibration was based on the three peaks always present in the spectra: H_3_O^+^ (*m/z* 19.0178), ^15^NO^+^ (*m/z* 30.9945), and NO_2_^+^ (*m/z* 45.9924).

The transmission factors of the ions under study (parameters necessary to correct discrimination of ions caused by variation in the extraction efficiency of ions from the drift tube, the transmission efficiency of the TOF filter and the detection efficiency of the electron multiplier) were calculated using an instrument-specific transmission curve. This curve was built on the basis of several measured transmission factors, which had been experimentally determined using a multi-compound gas standard containing compounds with molecular weights ranging from 21 to 180. In practice, the transmission curve and the particular transmission factors of the ions under study were calculated using an algorithm implemented in the software provided by the manufacturer (PTR-MS Viewer 3.1.0).[51]

The standard mixtures were introduced into the flow/drift tube of the SRI-TOF-MS instrument at a steady flow rate of 10 mL/min via a 2-m-long, heated (40 °C) Teflon transfer line. The total duration of a single measurement was 5 min, which corresponds to 10 mass spectra acquired per concentration (ppbv) level. Effectively, the average of these 10 spectra was used to determine the percentages of the product ions resulting from each NO^+^/organosulfur (NO^+^/organoselenium) compound reaction.

## RESULTS AND DISCUSSION

Table 2 lists the product ion distributions, in percent, for the reactions of NO^+^ with the 15 volatiles under study. These percentages were calculated using the signal intensities corrected by the transmission factors calculated from the discrimination function. The product ion percentages given are the averages of values obtained for three distinct mean concentrations (from 10 spectra) of the volatile analytes in the dry air and the humid air samples. Only product ions with abundance greater than 0.5% of the total signal were included in Table 2 unless they clearly originated from the species under study. Nevertheless, the given product ions percentages should be considered in the light of the purities of each compound as given in Table 2.

Under the conditions of this particular SRI-TOF-MS instrument at a drift field intensity of 130 Td, the dominant reaction mechanism of the NO^+^/VSCs reactions is charge transfer generating M^+^ ions. These finding shows that the ionization energies (IEs) of the compounds under study are smaller than the recombination energy of the reactive NO^+^ nitrosonium ion (which for its ground rovibrational state is 9.26 eV). This is in accordance with earlier studies carried out under the thermalised condition of SIFT-MS.[18,52] Strikingly, methanethiol was mainly ionized via charge transfer despite the fact that its IE (9.46 eV) is greater than that for the ground rovibronic state of NO^+^. This implies that the NO^+^ ions are vibrationally/kinetically excited during their formation in the ion source and that a fraction of these excited ions persists in the drift tube. On the other hand, the methanethiol/NO^+^ reaction results also in the production of a small fraction of the adduct ion [CH_3_ SHNO]^+^. The presence of this adduct ion is evidence for a distribution amongst the NO^+^ vibrational states, with a fraction of these ions being in the lowest vibrational state; these low vibrational state ions are known from SIFT studies to be prone to undergo association reactions with compounds, such as several ketones, with similar IEs to NO.[53] The reactions of NO^+^ with methanethiol and allyl isothiocyanate are complicated since, in addition to M^+^ and [M NO]^+^ products, significant fractions of [M–H]^+^ ions are produced via hydride ion transfer and these would have to be accounted for in quantitative analysis using this particular SRI-TOF-MS setup. Interestingly, in the reactions of NO^+^ with dimethyl sulfoxide, the hydroxide ion transfer [R–OH]^+^ product ion was also observed, but the percentage of this channel was small at 6%.

In the majority of these VSC reactions with NO^+^ the dominant product ion is the M^+^ cation that comprises more than 85% of the total product ions. The two exceptions to this are the ethyl methyl sulfide and methyl propyl sulfide reactions for which the fractions of the M^+^ are 42% and 22%, respectively:


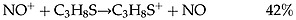
(1a)


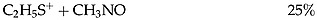
(1b)


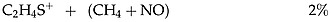
(1c)


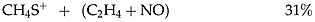
(1d)

The elimination of CH_3_^•^, exemplified by reaction (1b) is seen to be a common fragmentation channel, probably leading to CH_3_NO (isomer of formamide), but positive identification of the neutral products of the observed fragmentation reaction channels remains ambiguous, as is indicated by the bracketing of most of them in Table 2. For instance, the ejection of CH_3_ in the dimethyl sulfide reaction could produce separated CH_3_ and NO, or the single neutral molecules HCONH_2_ (formamide) or CH_3_NO. The production of the separated species CH_3_ and NO can be shown to be endothermic by 184 kJ/mol[54] and is thereby very unlikely. The formation of a formamide molecule is exothermic (by 240 kJ/mol[54]), but it would require some rearrangement within the nascent NO^+^/dimethyl sulfide complex. The production of CH_3_NO is slightly endothermic by 16 kJ/mol; however, it could be driven by the elevated interaction energies prevailing in this particular SRI-TOF-MS instrument at an E/N of 130 Td. Moreover, in the case of some heavier sulfides such as ethyl methyl sulfide or dimethyl disulfide, the production of CH_3_NO is clearly exothermic by 43 and 64 kJ/mole, respectively.[54] Nevertheless, these observed product channels represent just small fractions of the total product ions (<5%) and so would not greatly affect quantification if they were not considered in the analysis of these compounds using this particular SRI-TOF-MS instrument operated at 130 Td. The higher degree of fragmentation in the ethyl methyl sulfide and methyl propyl sulfide reactions can be ascribed to the presence of the longer alkane chain in the sulfide molecule. Interestingly, the presence of an unsaturated hydrocarbon chain seems to prevent fragmentation. For instance, the percentage of the M^+^ ion product in the methyl propyl sulfide/NO^+^ reaction is 20-25%, whereas, for the allyl methyl sulfide/NO^+^ reaction it is more than 85%. It must be stressed here that some of the observed low percentage fragmentation channels could stem from the impurities in the liquids used for the generation of the standards (e.g. C_2_H_6_S^+^ ion in the dimethyl trisulfide spectrum might be evidence for the presence of the dimethyl sulfide impurities) or be caused by partial thermal dissociation (or metal-catalyzed reactions) along the sample flow lines and the heated reactor tube. It is difficult to completely eliminate these effects in studies dealing with such a reactive class of compounds.

Analogously to VSCs, both the volatile selenium compounds included in this study reacted with NO^+^ via charge transfer and showed very little fragmentation. The only observed fragmentation channel was very weak (<1%) and related to CH_3_ radical elimination. These findings are essentially in agreement with earlier studies on the selenium compounds/NO^+^ reactions performed under the thermalized condition of a SIFT-MS instrument.[35] An interesting point is that, despite the single ionization mechanism and the absence of fragmentation, the product ion distributions of these NO^+^/selenium reactions are relatively complex. This is because the selenium atom has seven naturally occurring stable isotopes. This is particularly pronounced when two selenium atoms are incorporated into the molecule, as in case of dimethyl diselenide. To illustrate this effect, the percentages of the observed main isotopologue charge transfer product ions are shown in Table 2; these relative abundances of the isotopologue ions are in good agreement with the calculated values based on the isotopic abundances.[35] Such a pattern formed by isotopologue ions might be used as a fingerprint of selenium compounds, which is very useful for their identification and detection in complex samples as long as serious overlaps with isobaric ions from other reactive compounds in the sample mixture can be identified and accounted for.

The presence of water molecules in the sample has little effect on the product ion distributions. However, the major product ion of the NO^+^/methanethiol reaction, CH_3_SH^+^, overlaps with a large, nominally isobaric, [H_2_O NO]^+^ adduct ion formed when water molecules are present in the SRI-TOF-MS reactor, the abundance of which increases considerably when humid samples are involved. Consequently, the utility of the CH_3_SH^+^ ion for the identification and quantification of methanethiol can be problematic, but by exploiting the high-resolution TOF analyser these two ions can be separately identified and quantified as long as the relatively higher NO^+^H_2_O ion signal does not significantly overlap with the much smaller CH_3_SH^+^ ion signal. If this fails, methanethiol analysis can be successfully achieved using H_3_O^+^ precursor (analyte) ions as has been demonstrated in recent SIFT-MS studies.[21,22]

## CONCLUDING REMARKS

The results of the present study indicate that most of the reactions of NO^+^ with volatile organosulfur and organoselenium compounds result in the formation of the M^+^ product ions resulting from exothermic charge transfer. The two exceptions are ethyl methyl sulfide and methyl propyl sulphide, for which M^+^ ions are not as dominant and channels are seen that indicate the elimination of neutral CH_3_, CH_4_ and/or C_2_H_4_ species that are probably bound to NO molecules, as is suggested by the bracketing of neutral products of reactions in Table 2. Significant fractions of the ion-molecule association adduct ion [M NO]^+^ and the hydride ion transfer ion [M–H]^+^ were also observed in the NO^+^/methanethiol and NO^+^/allyl isothiocyanate reactions. There are several other fragmentation channels in most of the reactions, but they usually represent only small fractions of the total product ions.

The rationale for these product ion distribution studies was to identify the major analyte (product) ions of the NO^+^ reactions and thus to prepare the way for the analysis by SRI-MS of these organosulfur and organoselenium compounds present in exhaled breath, in emanations from skin, and in the headspace of urine, blood and cell and bacterial cultures. For such analyses the reaction rate constants for the reactions must also be known, but these were not determined in the present experiments. However, all the reactions studied are probably exothermic and proceed at the gas kinetic (collisional) rate (that can be derived by calculation[55]). This was certainly seen to be the case in an earlier SIFT study of the reactions of NO^+^ with several other volatile organosulfur compounds.[52] Thus, in general, the gas kinetic rate constants for these reactions can be used with confidence for analysis, although the suprathermal conditions of SRI-MS reactors may modify the rate constants somewhat from their respective thermal gas kinetic values. This is implicit in what has been said previously in that the value of E/N set for these ion chemistry experiments, and by inference the values of E/N used for gas-phase analysis, will surely modify to some extent the rate coefficients for the reactions and the product ion distributions. This is certainly the case for many NO^+^/ketone reactions in which adduct ion formation is the dominant process.[56] Thus, for accurate analyses, the rate constants and product ions must be determined at the chosen E/N value or standard mixtures used to 'calibrate' the analytical instrument, although the latter approach is challenging when 'sticky' compounds such as these organosulfur and organoselenium compounds are involved.
